# MicroRNA profile comparison of the corneal endothelia of young and old mice: implications for senescence of the corneal endothelium

**Published:** 2013-08-06

**Authors:** Xiaowen Zhao, Yusen Huang, Ye Wang, Peng Chen, Yang Yu, Zicheng Song

**Affiliations:** State Key Laboratory Cultivation Base, Shandong Provincial Key Laboratory of Ophthalmology, Shandong Eye Institute, Shandong Academy of Medical Sciences, Qingdao, China

## Abstract

**Purpose:**

To identify critical microRNAs (miRNAs) that play important roles in regulating the aging of corneal endothelial cells in mice aged 10–13 weeks and 2 years.

**Methods:**

We collected the corneal endothelia from 30 mice aged 10–13 weeks and 30 mice aged 2 years. The samples were pooled into six groups (Y1, Y2, Y3 and S1, S2, S3). Each group comprised corneal endothelia from 10 mice, and these six groups were used for a genome-wide miRNA microarray study. The expression levels of eight selected miRNAs were further validated independently by quantitative reverse transcription polymerase chain reaction (qRT-PCR). Target genes were predicted using a computational approach due to their base-pairing rules between miRNA and messenger RNA target sites. The locations of binding sequences were within the target’s 3′ untranslated regions (UTR), and the conservation of target binding sequences occurred within related genomes. Additional gene ontology and signaling pathway analyses were performed using bioinformatics tools.

**Results:**

Twenty-seven miRNAs (7 upregulated and 20 downregulated) were found to be differentially expressed (fold change >2, p value <0.05) in the corneal endothelia of adult and old mice. The qRT-PCR results confirmed the differential expression of eight miRNAs between the corneal endothelia of adult and old mice. A computational approach demonstrated that the target genes of the differentially expressed miRNAs might be involved in several signaling pathways, including the glutamatergic synapse pathway (p=0.000313), the phosphatidylinositol signaling pathway (p=0.00197), the neurotrophin signaling pathway (p=0.00687), the transforming growth factor–beta signaling pathway (p=0.0143), and oxidative phosphorylation (p=0.0223).

**Conclusions:**

Our study identified miRNAs that are differentially expressed in the corneal endothelium during aging for the first time. We also identified fluctuations in the expression of these specific miRNAs that may be related to age-specific changes. Understanding miRNA expression and interactions in tissues such as the cornea may aid in the understanding of the basic and pathophysiological processes of age-related ocular pathologies.

## Introduction

The normal human corneal endothelium is a monolayer of uniformly sized cells in a well-arranged mosaic pattern. The most important physiological function of the corneal endothelium is regulation of the water content of the corneal stroma. Therefore, corneal endothelial cells are the key cell type in maintaining corneal clarity via the regulation of corneal hydration [1]. Despite its physiological importance, however, the corneal endothelium is an extremely fragile tissue because of its inability to divide and proliferate. Aging, trauma, intraocular surgery, and many diseases, including corneal opacification following cataract surgery, keratoconus, inherited disorders, and scarring caused by infections, can cause changes in the endothelial monolayer. Studies have shown that human corneal endothelial cells (HCECs) are arrested in the G1 phase of the cell cycle and do not proliferate ex vivo [2]. Due to this relative lack of cell division, HCEC density in the normal, healthy cornea decreases with age [3-5]; however, the reason for this remains unclear. Studies in our laboratory have shown an age-related increase in *p16^INK4a^* expression in the corneal endothelium of both humans [6] and senescence-accelerated mice [7] in vivo. Many studies have shown that cell senescence is closely related to the expression of the inhibitor of cyclin-dependent kinase, *p16^INK4a^*, which is also a biomarker of tissue aging. Furthermore, *p16^INK4a^* plays a decisive role in regulating G1 arrest [8-10]. Therefore, this senescent state of corneal endothelial cells is hypothesized to severely restrict proliferation in vivo; thus, a better understanding of the regulatory mechanisms underlying the senescence of corneal endothelial cells should lead to the improved management of age-related corneal pathologies.

MicroRNAs (miRNAs) are small, noncoding RNAs that play important roles in the regulation of target genes. These miRNAs have been correlated with several diseases, such as diabetes, neurodegenerative diseases, and heart failure [11-13], and bind to complementary regions of messenger transcripts to either repress translation or regulate degradation [14,15]. Recent miRNA expression profiling tools have facilitated the detection of miRNAs, making it easier to identify the functional role of miRNAs in different tissues and species in both physiological and pathological processes. Currently, several reports are available regarding the expression profile of miRNAs in the eye. Most of these publications analyze the miRNAs in the retina, cataract lens, and corneal epithelium [16,17], particularly in terms of disease. Little is known about age-related miRNA expression in the cornea, particularly in the corneal endothelium. In this study, we performed miRNA profiling of BALB/c mice at 10–13 weeks and 2 years of age to obtain a global view of differential miRNA expression in the corneal endothelium. The expression of selected miRNAs was further validated independently by quantitative reverse transcription polymerase chain reaction (qRT-PCR). Target genes were predicted using the miRNA prediction algorithms TargetScan, miRanda, and PicTar. Additional gene ontology and signaling pathway analyses were performed using bioinformatics tools.

## Methods

### Animals and sample collection

BALB/c mice were obtained from the Institute of Zoology, Chinese Academy of Sciences, Beijing, China. Mice aged 10–13 weeks were used as adult mice, and mice aged 2 years old were used as old mice. The Institutional Animal Care Committee approved this study, and all procedures were performed in accordance with the Association for Research and Vision in Ophthalmology Statement for the Use of Animals in Ophthalmic and Vision Research. The corneas were trephined with a diameter of 2 mm and were placed endothelial side up on a Teflon block under a surgical microscope. Descemet’s membrane was easily detached from the underlying stroma with forceps and was stripped away intact with the endothelium. The stripped endothelial tissues were frozen at −80 °C for RNA analysis. We collected the corneal endothelia from 30 mice aged 10–13 weeks and from 30 mice aged 2 years. The samples were pooled into six groups (Y1, Y2, Y3 and S1, S2, S3), each of which comprised corneal endothelia from 10 mice; and these six groups were used for a genome-wide microRNA microarray study.

### RNA extraction and microRNA microarray study

Total RNA was isolated using the NucleoSpin RNA II kit (Macherey-Nagel, Düren, Germany) according to the manufacturer’s instructions. Briefly, the samples were lysed by incubation in a solution provided with the kit. After lysis, homogenization and reduction of viscosity are achieved by filtration with NucleoSpin® Filter units provided with the kit. Total RNA is finally eluted with RNase-free water supplied with the kit. MicroRNA microarray analysis, including labeling, hybridization, scanning, normalization, and data analysis, was performed by KangChen Bio-tech (Shanghai, China) on a miRCURY LNA microRNA array (v.16.0, Exiqon, Vedbaek, Denmark). The miRCURY Hy3/Hy5 Power labeling kit (Exiqon) was used according to the manufacturer’s instructions for miRNA labeling. After the labeling procedure was completed, the Hy3-labeled samples were hybridized to the miRCURY LNA Array according to the array instructions. The arrays were then washed and immediately scanned using a GenePix 4000B array scanner (Axon Instruments, Foster City, CA).

### Analysis of microarray data

The scanned images were imported into the GenePix Pro 6.0 software (Axon Instruments) for grid alignment and data extraction. Replicated miRNAs were averaged, and the miRNAs with intensities ≥50 in all samples were chosen to calculate the median normalization factor. The expressed miRNA data were normalized using the median normalization and chosen for the differentially expressed miRNA screening. To identify differentially expressed miRNAs with statistical significance, we performed a Volcano plot filtering between the two groups of the experiment. The threshold we used to screen for up- or downregulated miRNAs was a fold change ≥1.5 and a p value ≤0.05. A false-discovery rate less than 5% was considered significant in this study.

### MicroRNA target gene prediction and functional analysis

Prediction of miRNA target genes can be performed using a computational approach. First, the potential binding sites in the messenger RNA 3′ according to specific base-pairing rules were identified, and second, implementation of cross-species conservation requirements was performed. The prediction of miRNA target genes was carried out with the following three different miRNA target prediction algorithms: PicTar [18], miRanda v5 [19], and TargetScan v5.1 [20]. Based on these database searches, the genes with target sites for all of three coexpressed miRNAs were identified as a potential cooperative target gene set. We also used the Matchminer program [12,21] to determine the genes that were identified by at least two algorithms. Then, these results were integrated into the gene network analysis using the Medusa software program [22].

We identified the significant Gene Ontology (GO) classifications and Kyoto Encyclopedia Genes and Genomes pathways using the DAVID Bioinformatics Resources [23]. Fisher’s exact test was used to determine the enrichment in categories with target genes in the DAVID bioinformatics resource.

### Quantitative reverse transcription polymerase chain reaction microRNA analysis

To validate the reproducibility of the results from the miRNA microarray, qRT-PCR analysis of (microRNAs come from mice) mmu-miR-695, mmu-miR-183, mmu-miR-182, mmu-miR-194, mmu-miR-34c, mmu-miR-31, mmu-miR-190, and mmu-miR-124 was performed using the same extracted total RNA as the microarray analysis. The first-strand cDNA was synthesized from equal amounts of total RNA using an MMLV Reverse Transcriptase 1st-Strand cDNA Synthesis Kit (Epicenter Biotechnologies, Madison, WI) according to the manufacturer’s protocol. All specific primers were designed and synthesized by Guangzhou RiboBio (RiboBio, Guangzhou, China) using the qRT-PCR Primer Sets (catalog no. MQP-0101). A specific primer for each miRNA used in reverse transcription was in these Primer Sets. Briefly, in a 20 μl reaction volume, 1 μM of each specific reverse transcription primer (0.3 μl), 2 μl dNTP (2.5mM; HyTest Ltd., Turku, Finland), 2 μl 10X RT buffer (Promega Corporation, Madison, WI), 20 U MMLV Reverse Transcriptase (10 U/μl; Epicenter Biotechnologies), 0.3 μL RNase inhibitor (40 U/μl), and 1 μg of total RNA was added.

qRT-PCR was performed using the SYBR Green protocol on an ABI 7500 system (Applied Biosystems, Foster City, CA), and a data analysis was performed with the SDS system software (7500 System; Applied Biosystems). The levels of an endogenous control, U6 (RiboBio, Guangzhou, China; catalog no. MQP-0201), were used to normalize the expression levels of each miRNA. All reactions were performed in triplicate and included controls without a template for each miRNA. The fold change in miRNA expression was calculated using the comparative Ct method. The corneal endothelium of a young mouse was used as a calibrator, and the data were presented as the fold change relative to the calibrator.

### Statistical analysis

All results were expressed as the mean±standard deviation (SD). A statistical analysis was performed with the Student *t* test to determine any significant differences using commercial software (SPSS 11.5; SPSS, Chicago, IL). A value of p<0.05 was considered statistically significant.

## Results

### Global differential microRNA profiling in the corneal endothelia of young and old mice

**Figure 1:**
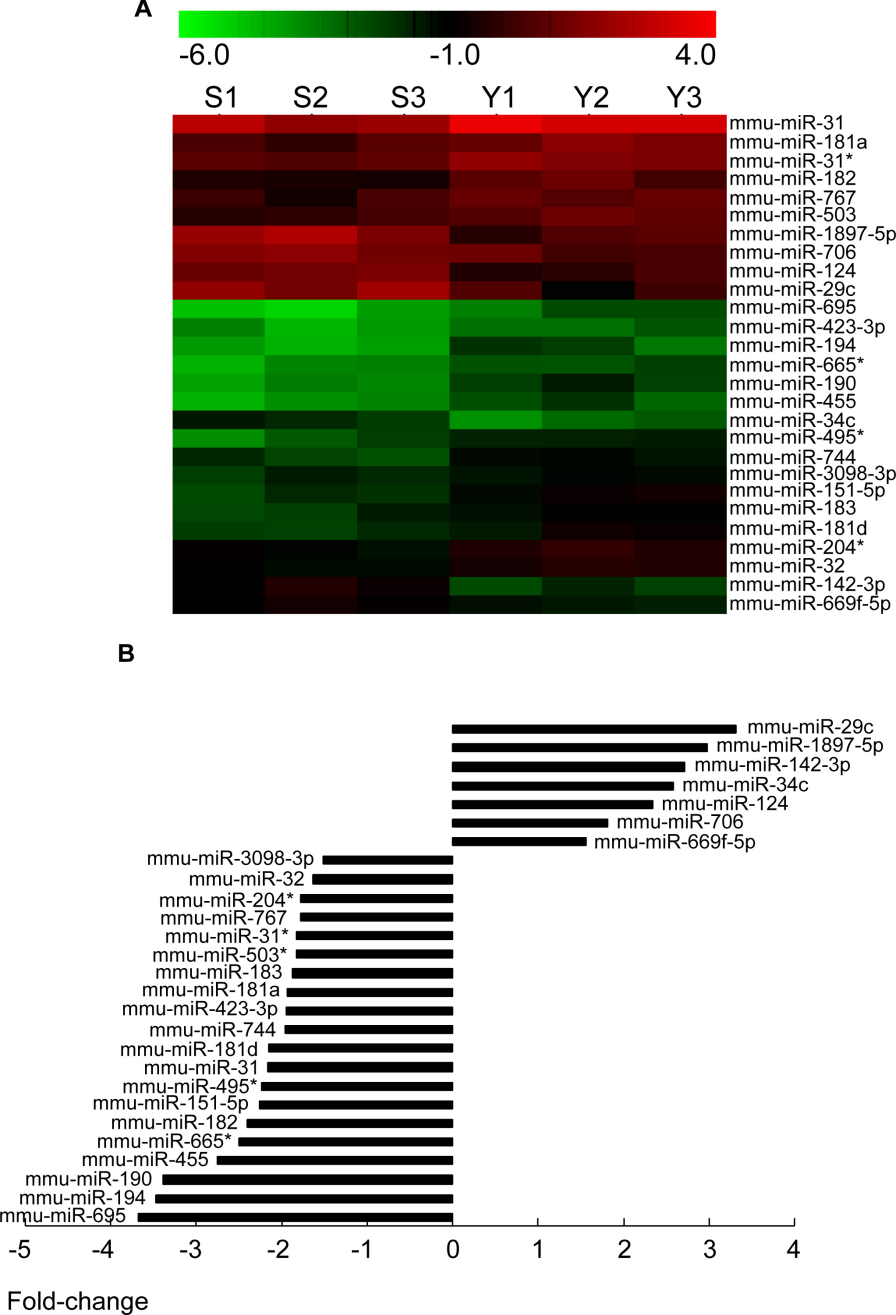
MicroRNA profiles were performed between the corneal endothelium of young and old mice. **A**: Hierarchical clustering was performed with normalized microRNA (miRNA) data (fold change >2) that passed the Student *t* test (p<0.05). A total of 27 miRNAs were identified whose expression was significantly altered in the corneal endothelium of young and old mice. Rows, miRNA; Column, the corneal endothelia of young mice (Y1, Y2, and Y3) and old mice (S1, S2, and S3). For each miRNA, red color indicates genes with high expression and green color denotes genes with low expression. **B**: Fold change (ratio between old/young animals) in miRNA expression between the corneal endothelium of young mice and old mice. The corneal endothelia of young mice were used as control. The fold change ranged from −3.31 to −1.56 and from 1.53 to 3.67 for the upregulation and downregulation of miRNAs, respectively. Mmu-miR-29c exhibited the greatest decrease in expression, whereas mmu-miR-695 exhibited the greatest increase in expression in the old mice.

**Figure 2:**
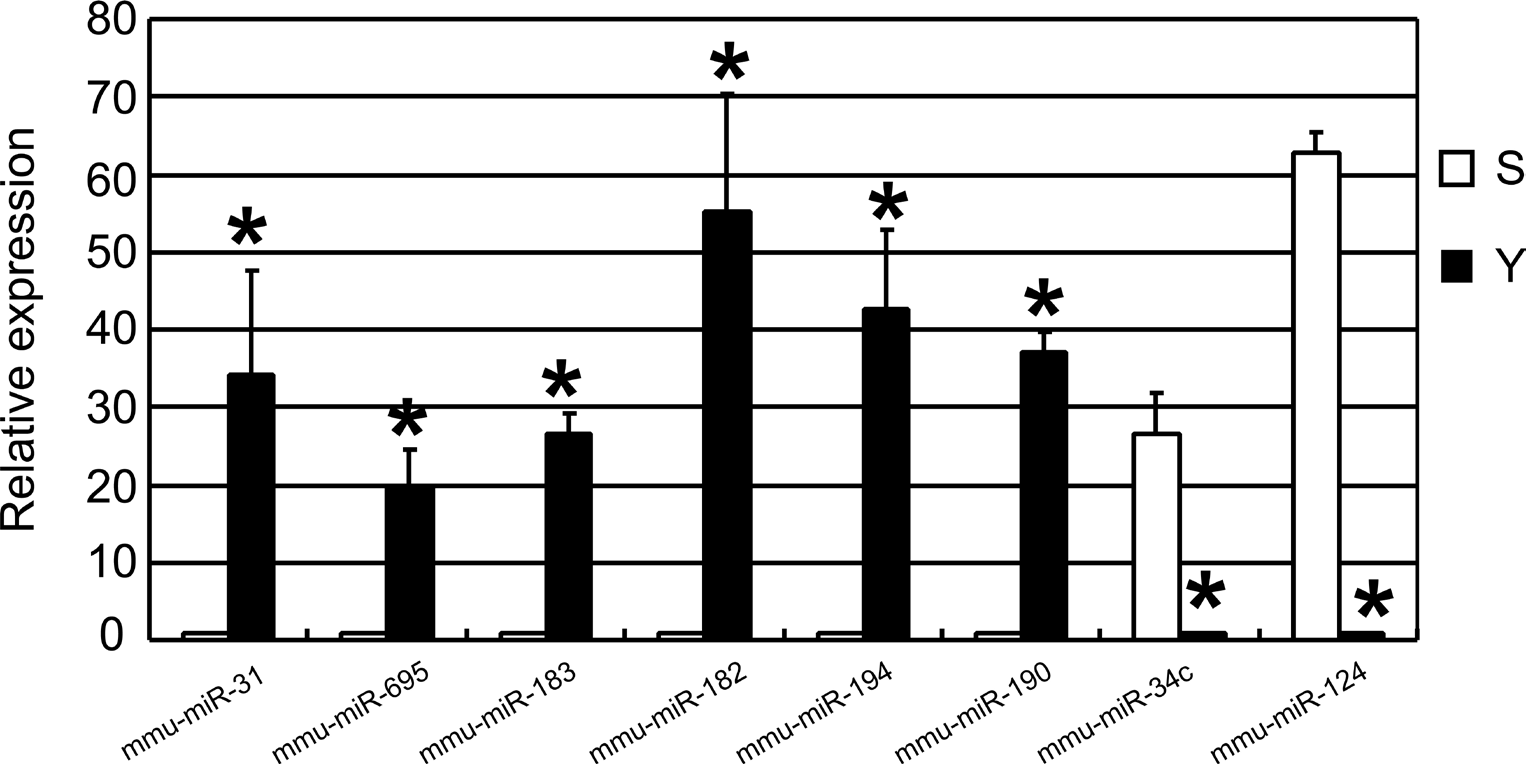
Validation of selected microarray data by quantitative reverse transcription polymerase chain reaction. Relative expression levels of selected microRNAs (miRNAs) were determined by quantitative reverse transcription polymerase chain reaction (qRT-PCR). The results showed that the expression of mmu-miR-31, mmu-miR-695, mmu-miR-183, mmu-miR-182, mmu-miR-194, and mmu-miR-190 markedly downregulated in the corneal endothelium of old mice compared to young mice. Meanwhile, the expression of mmu-miR-34c and mmu-miR-124 markedly upregulated in the corneal endothelium of old mice compared to young mice. Values are mean±standard deviation (SD) and expressed relative to internal control (U6; n=3 for each group, * p<0.05, Student *t* test).

To identify miRNAs that might be involved in senescence of the corneal endothelium, we assessed the miRNA expression profiles in the corneal endothelia of young and old mice using the miRCURY LNA Array platform. We collected the corneal endothelia from 30 mice aged 10–13 weeks and the corneal endothelia from 30 mice aged 2 years. The samples were pooled into six groups (Y1, Y2, Y3 and S1, S2, S3; n=3 in each group). Each group comprised corneal endothelia from 10 mice, and the six groups were used for a genome-wide microRNA microarray study. For the S groups, the amount of RNA obtained from 10 corneal endothelia was 56.6 μg (concentration=2.83 μg/μl; volume=20 μl; OD260/280 Ratio=2.08; the ratio of the absorbance at 260 and 280nm (A260/280) is used to assess the purity of nucleic acids. For pure RNA A260/280 is ~2). For the Y group, the amount of RNA obtained from 10 corneal endothelia was 63.2 μg (concentration=3.16 μg/μl; volume=20 μl; OD260/280 Ratio=2.07). The miRNA expression in the corneal endothelium of young (n=3) and old mice (n=3) was initially determined through three separate microarray assays. Full lists of normalized microRNA expression are shown in supplemental Table 1 and Table 2. Based on the results of a Student *t* test, we carried out Volcano plot filtering between the two groups. The threshold we used to screen up- or downregulated miRNAs was a fold change ≥1.5 and a p value ≤0.05. A total of 160 miRNAs (15% of 1,055 miRNAs represented on the array) were found to be differentially expressed, and only 27 miRNAs passed the Volcano plot filtering screen at the significance level (p<0.05, false discovery rate <0.05, Figure 1A). Among these miRNAs, 20 were found to be downregulated less than 0.5-fold, and 7 were found to be upregulated more than 1.5-fold in the corneal endothelia of old mice compared to young mice (Figure 1B). The fold change ranged from −3.31 to −1.56 and from 1.53 to 3.67 for the old and young mice, respectively. Mmu-miR-29c exhibited the greatest increase in expression, whereas mmu-miR-695 exhibited the greatest decrease in expression in the old mice. A full list of normalized microRNA expression was shown as a supplement. We then used qRT-PCR to validate the expression of the eight selected miRNAs. Mmu-miR-695, mmu-miR-31, mmu-miR-190, mmu-miR-183, mmu-miR-182, and mmu-miR-194 were the most significantly downregulated miRNAs, whereas mmu-miR-34c and mmu-miR-124 were the most significantly upregulated miRNAs. The qRT-PCR results demonstrated a decrease in the expression of mmu-miR-31(34.2±13.4-fold), mmu-miR-695 (19.8±4.79-fold), mmu-miR-183 (26.6±2.53-fold), mmu-miR-182 (55.2±15.3-fold), mmu-miR-194 (42.6±10.2-fold) and mmu-miR-190 (37.1±2.78-fold) in the corneal endothelium of old mice compared to young mice, whereas the expression of mmu-miR-34c and mmu-miR-124 increased 26.4±5.28-fold and 62.7±2.54-fold, respectively (Figure 2). These data were consistent with the microarray results.

**Table 1:** The results of the target gene and network analysis of selected miRNAs in the corneal endothelium of old mice compared to young mice.

**miRNA_name**	**Gene symbol**
mmu-mir-181a	2310067B10RIK, 5730419I09RIK, A930001N09RIK, ADHFE1, ARSJ, ATP6V1A, ATXN1, CBX4, CCAR1, CPD, CUL3, DDIT4, DDX3X, DHX57, DLGAP2, DOCK7, EED, EIF4A2, ELAVL4, ELP4, ESM1, EVI2A, HAND2, HSP90B1, IL1A, ILF3, INPP5E, KLHL5, LYRM1, MARK1, NNT, NPTN, NR6A1, OSBPL3, PAM, PDAP1, PHLDA1, PHOX2B, PLEKHJ1, PRKCD, RALA, RASSF8, RBBP7, RCBTB2, GMA, RNF145, SCHIP1, SEMA4C, SHOC2, SIN3B, SIX2, SLC4A10, SLC9A6, SYT3, TANC2, TXNDC12, ZBTB4, ZFAND6, ZMYND11
mmu-mir-181d	2610305D13RIK, 5730528L13RIK, AFG3L2, CPNE2, E2F5, FIGN, GDI1, HEY2, HMBS, IGF2BP2, LRRN1, MLF1, MOSPD1, MTAP1A, NDRG2, NPEPPS, PAK4, RASSF1, SEMA4G, TBC1D4, TOX, WDR20A,
mmu-mir-182	AADAT, AATK, ABHD2, ACVR1C, ADAM22, ADRA2C, AMPH, ANK3, ANXA11, ARF4, ARHGAP29, BDNF, CCDC41, CDO1, CDSN, CEP250, CHL1, CLPTM1L, COBL, CORO1C, CREB3L1, CTDSP1, CTTN, DAZAP2, DCUN1D4, DOS, EPAS1, EPHB1, ETL4, EVI5, EXOC4, FLOT1, FMR1, FOXF2, FOXO3, GNA13, HAND1, INPP5A, ISL1, JAZF1, KCMF1, KDELR1, KRT84, KTN1, L1CAM, LPHN2, LSM14A, MAGEL2, MARCKS, MGAT4C, MOBKL1A, MTCH2, MYO1B, NCOA4, NPM1, NRN1, OLFR976, PAIP2, PCDH8, PCX, PDIA4, PDZD4, PEX5, PLD1, PNPLA2, PPIL1, PPP1R13B, PPP1R2, PRDM1, RAB19, RAC1, RARG, RASA1, RGS17, RTN4, SH2D1A, SH3BP4, SLC1A2, SLITRK4, SLMO2, SNAP23, SP3, SPIN1, STK19, STK36, STOX2, TAF4A, TGFBI, TMEM115, TOB1, TOPBP1, TSNAX, WDR47, WHSC1L1, WWC2, XBP1, ZFP36, ZMPSTE24
mmu-mir-183	GMFB, GNB1, GNG5, HN1, IDH2, ITGB1, KCNK10, KCNK2, KIF2A, L3MBTL3, LRP6, MAPK8IP1, MTMR6, NPC2, PCDHGA8, PDCD4, PDCD6, PKP4, PLEKHA3, PPP2CB, PPP2R5C, PSEN2, PTDSS1, PTPN4, RCN2, RNF138, SEL1L, SLC35A1, SLITRK1, SLITRK3, 1810013L24RIK, AI314180, ARHGAP21, ASH2L, BC030476, BRD4, BTG1, CLCN3, CSMD1, DUSP10, FCHO2, SNX1, SOBP, SPCS2, SPRY2, STK38L, TMPO, TPM1, TTC14, UNC13B, ZDHHC6, ZEB2, ZFP592, ZFP609, ZFPM2, ZMYM2
mmu-mir-190	AHSA2, BC052040, BDNF, CEBPA, FGF14, GPHN, LMCD1, NKX6–1, SAMD4, SETBP1, SLC17A6
mmu-mir-31	1700066B19RIK, ACTG1, AF529169, AHSG, ATP8A1, COL5A1, COPS2, CTNND2, FNDC5, GLTSCR1, LYZL6, MAP4K5, MBOAT2, OSBP2, POU2F3, PPP3CA, PPP6C, SH2D1A, SLC35A2, SUPT16H, TACC2, TAF4A, TMPRSS11F, TRP53INP2, VPS39
mmu-mir-32	B3GALT2, ARHGAP29, BCL11B, CCNC, CCNL2, CHKA, COL27A1, DCBLD1, DKK3, DNAJB9, DPP10, FRY, GAP43, GFPT2, HAND2, HAS2, HERC2, HERPUD2, HIVEP1, HPS6, IBSP, ITGA6, ITPR1, KALRN, KIF5B, KLF2, LPIN1, LRRC4, LRRC8D, MORC3, MYO1B, PAX9, PCMTD1, PCOLCE2, PIK3CB, PPCS, PPP1R12C, PTPRK, RAB14, RSBN1, SLC25A32, SLC32A1, SMAD6, SMAD7, SUV420H1, SYNJ1, TFDP2, TSGA14, UGP2, USP28, ZFYVE21
mmu-mir-455	ABCF3, ANKS4B, ARMC8, BAX, BRPF1, D10WSU52E, DLG4, FBXL15, H13, H2AFX, HOXC4, HSF1, LHX2, MAGI1, MOSPD1, NDUFA2, NR2F2, PAX6, PCBP2, PCDH9, PLCD4, PNCK, PPP1R10, RMND5A, RTN4, RUSC1, SAP130, SCUBE2, SLC35F1, SMTN, SNX2, SSR1, TMEM62, TRAF1, TRIM3, VSNL1, ZFP238
mmu-mir-695	EIF2S1, EIF4E, SSBP3
mmu-mir-744	LRP3, NRGN, PPFIA3, RARA, SH3BGRL3, VPS37D
mmu-mir-706	ARHGEF17, ATF7IP2, ATP5G1, CEPT1, COPB1, CYC1, DACH2, H2AFV, HOXD13, MLH3, PABPC4, PBX1, PTGS1, STX8, TAF4A, TIA1, TNP1, TRIP12, WIPF2
mmu-mir-29c	ADAMTS18, COL4A1
mmu-mir-34c	NFE2L1, STK38L

The selected miRNAs were mmu-miR-181a, mmu-miR-181d, mmu-miR-182, mmu-miR-183, mmu-miR-190, mmu-miR-31, mmu-miR-32, mmu-miR-455, mmu-miR-695, mmu-miR-744, mmu-miR-706, mmu-miR-29c, and mmu-miR-34c. Prediction of miRNA target genes can be performed by a computational approach. First, the potential binding sites in the mRNA 3′ according to specific base-pairing rules were identified and second, implementation of cross-species conservation requirements was performed. The prediction of miRNA target genes was performed with the following three different miRNA target prediction algorithms: PicTar, miRanda v5 and TargetScan v5.1. Each algorithm has a definite rate of both false positive and false negative predictions. Based on these database searches, the genes with target sites for all of three co-expressed miRNAs were identified as a potential cooperative target gene set. Then, these results were integrated into the gene network analysis using the software Medusa. The common target gene between mmu-miR-181d and mmu-miR-455 was motile sperm domain containing 1 (MOSPD1), that between mmu-miR-31 and mmu-miR-182 was RNA polymerase II, TATA box binding protein (TBP)-associated factor (TAF4A), that between mmu-miR-455 and mmu-miR-182 was reticulon 4 (RTN4), that between mmu-miR-182 and mmu-miR-190 was brain-derived neurotrophic factor (BDNF), that between mmu-miR-142–3p and mmu-miR-34c was protein phosphatase 1, regulatory subunit 10 (PPP1R10), and that between mmu-miR-142–3p and mmu-miR-124 was leucine rich repeat containing 1 (LRRC1).

**Table 2:** The results of Kyoto Encyclopedia Genes and Genomes (KEGG) Pathway assay.

PathwayID	Definition	Fisher-P value	Enrichment Score
mmu04724	Glutamatergic synapse	0.00031325	3.504109
mmu05200	Pathways in cancer	0.000364143	3.438728
mmu04360	Axon guidance	0.000371743	3.429757
mmu04070	Phosphatidylinositol signaling system	0.001969461	2.705653
mmu00562	Inositol phosphate metabolism	0.002646183	2.57738
mmu04666	Fc gamma R-mediated phagocytosis	0.004516641	2.345184
mmu05100	Bacterial invasion of epithelial cells	0.006824978	2.165899
mmu04722	Neurotrophin signaling pathway	0.006869456	2.163078
mmu04350	TGF-beta signaling pathway	0.01427558	1.845406
mmu05014	Amyotrophic lateral sclerosis (ALS)	0.01455702	1.836928
mmu04510	Focal adhesion	0.0197738	1.70391
mmu04721	Synaptic vesicle cycle	0.02158243	1.6659
mmu04141	Protein processing in endoplasmic reticulum	0.02556368	1.592377
mmu04530	Tight junction	0.02830684	1.548109
mmu04810	Regulation of actin cytoskeleton	0.03108982	1.507382
mmu05212	Pancreatic cancer	0.03178773	1.497741
mmu05211	Renal cell carcinoma	0.03323187	1.478445
mmu04725	Cholinergic synapse	0.04785029	1.320115
mmu00564	Glycerophospholipid metabolism	0.04976589	1.303068
mmu00190	Oxidative phosphorylation	0.02234092	1.650899

Kyoto Encyclopedia Genes and Genomes (KEGG) pathway analysis was performed to identify possible enrichment of genes with specific biologic themes on the basis of identify possible enrichment of genes with specific biologic themes on the basis of biologic process, cellular component, and molecular function. We identified the significant KEGG pathways using the DAVID Bioinformatics Resources. Fisher’s exact test was used to determine the enrichment in categories with target genes in the DAVID bioinformatics resource.

### Computational prediction of potential target genes and network analysis

As described above, candidate target genes for several up- or downregulated miRNAs were identified using three commonly used prediction algorithms—PicTar, TargetScan, and miRanda—to reduce the unpredictable number of false positives. The most significantly downregulated (mmu-miR-31, mmu-miR-455, mmu-miR-744, mmu-miR-695, mmu-miR-181a, mmu-miR-181d, mmu-miR-182, mmu-miR-190, mmu-miR-194) and upregulated miRNAs (mmu-miR-34c, mmu-miR-124, mmu-miR-142–3p, mmu-miR-706, mmu-miR-29c) were analyzed. The results of the target gene and network analysis are shown in Table 1. The pathway analyses showed that these target genes appeared to correlate with several pathways, such as the glutamatergic synapse pathway (p=0.000313), the phosphatidylinositol signaling pathway (p=0.00197), the neurotrophin signaling pathway (p=0.00687), the transforming growth factor–beta signaling pathway (p=0.0143), and oxidative phosphorylation (p=0.0223; Table 2). The results of the miRNA– messenger RNA regulatory networks indicated that the common target gene between mmu-miR-181d and mmu-miR-455 was motile sperm domain containing 1 (MOSPD1); that between mmu-miR-31 and mmu-miR-182 was RNA polymerase II, TATA box-binding protein–associated factor (TAF4A); that between mmu-miR-455 and mmu-miR-182 was reticulon 4 (RTN4); that between mmu-miR-182 and mmu-miR-190 was brain-derived neurotrophic factor (BDNF); that between mmu-miR-142–3p and mmu-miR-34c was protein phosphatase 1, regulatory subunit 10 (PPP1R10); and that between mmu-miR-142–3p and mmu-miR-124 was leucine rich repeat containing 1 (LRRC1).

### Microarray-based Gene Ontology analysis of differentially expressed microRNAs

**Figure 3:**
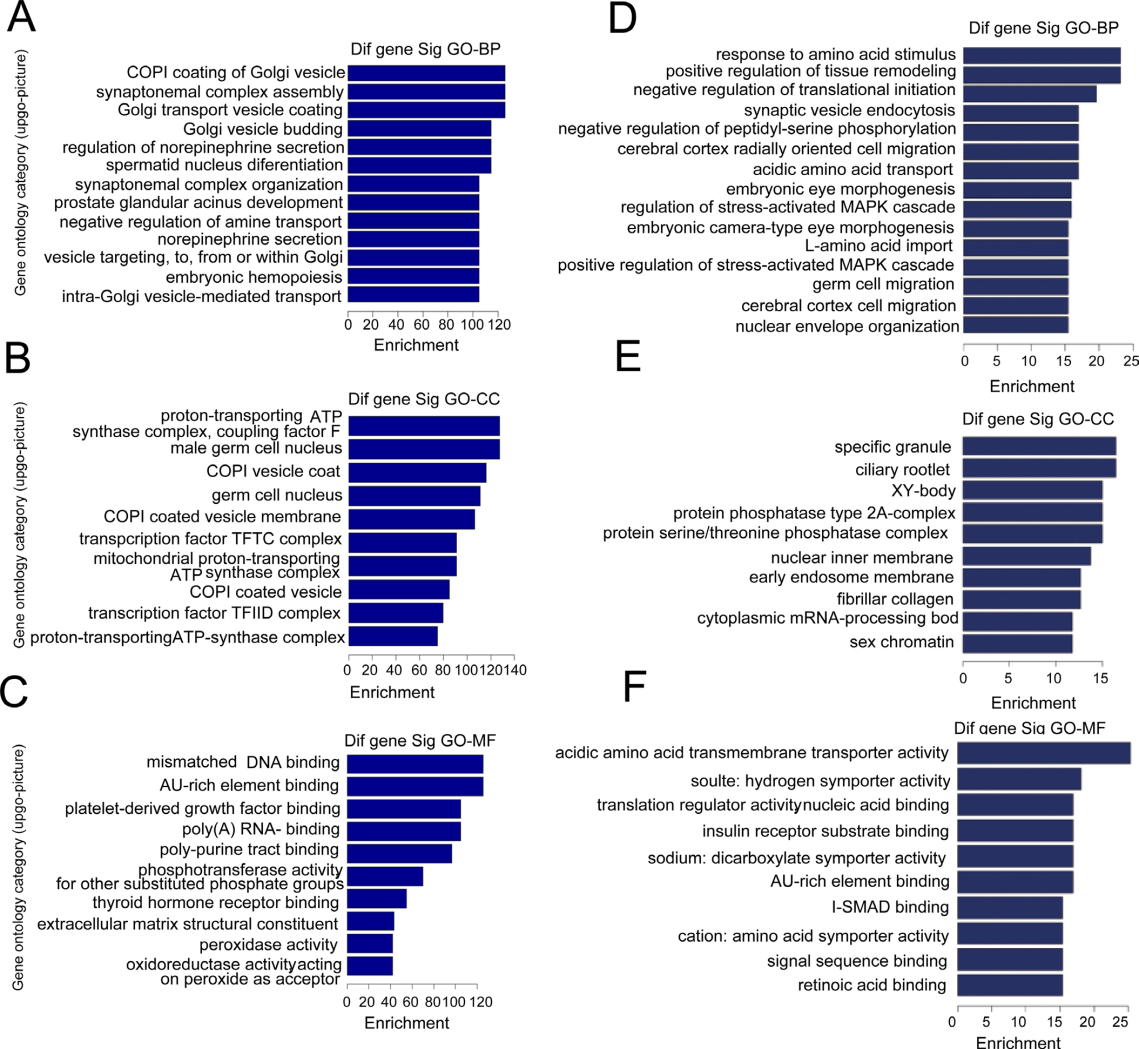
Microarray-based Gene Ontology analysis of differentially expressed microRNAs. **A**, **B**, and **C**: Gene ontology (GO) analysis of the most significantly upregulated microRNAs (miRNAs). **D**, **E**, and **F**: GO analysis of the most significantly downregulated miRNAs. The three GO classifications—molecular function (MF), biological process (BP), and cellular component (CC)—were evaluated separately and the significant terms of all ontologies are shown. The upregulated genes in the progressors were enriched in the coat protein (COPI) coating of Golgi vesicles (**A**), proton-transporting ATP synthase complex (**B**), and mismatched DNA binding (**C**), whereas downregulated genes in the progressors were enriched in tissue remodeling (**D**), stress-activated mitogen-activated protein kinase cascade (**E**), and insulin receptor substrate binding (**F**).

To gain insight into the biological roles of the most significantly down- or upregulated miRNAs, we performed a microarray-based GO analysis. Figure 3 shows the GO analysis of differentially expressed miRNAs. This analysis was performed to identify possible enrichment of genes with specific biological themes based on the three GO classifications of biological processes (BPs), cellular components (CCs), and molecular functions (MFs). MFs, BPs, and CCs, were evaluated separately, and the significant terms of all ontologies are shown. We identified the significant GO classifications using the DAVID bioinformatics resources. Fisher’s exact test was used to determine the enrichment in categories with target genes in the DAVID bioinformatics resource. The upregulated genes in the progressors were enriched in the coat protein (COPI) coating of Golgi vesicles (Figure 3A), proton-transporting ATP synthase complex (Figure 3B), and mismatched DNA binding (Figure 3C), whereas downregulated genes in the progressors were enriched in tissue remodeling (Figure 3D), stress-activated mitogen-activated protein kinase cascade (Figure 3E), and insulin receptor substrate binding (Figure 3F).

## Discussion

The corneal endothelium is a monolayer of neural crest–derived cells with limited regenerative potential, and these cells maintain stromal dehydration through an ion pump mechanism. Even with uncomplicated corneal transplantation surgery, endothelial cells are continually lost at an accelerated rate during the aging process. The fact that HCECs are arrested in the early G1 phase in vivo suggests that there is a degree of similarity between HCECs and senescent cells.

A study that aimed to identify the signaling pathways and mechanisms that contribute to both the proliferative lifespan and senescence in normal HCECs was undertaken. We previously reported the age-related increase in *p16^INK4a^* expression in normal HCECs in vivo [6] and in the senescence-accelerated mouse. The increased expression of *p16^INK4a^* is also an age-dependent phenomenon in the corneal endothelium [7]. Recently, we found that high expression of *p16^INK4a^* and low expression of *Bmi1* are associated with the cellular senescence of HCECs [24]. Sheerin et al. [25] characterized the cellular senescence mechanisms in human corneal endothelial cells. They evaluated the effects of exogenous human telomerase reverse transcriptase expression, p53 knockdown, disruption of the pRb pathway by overexpression of cyclin-dependent kinase 4 (CDK4) and reduced oxygen concentration on the lifespan of primary HCECs. The above-mentioned studies reported the common senescence phenotype and molecular mechanisms of normal HCECs; however, the mechanisms underlying corneal endothelial cell senescence in pathogenesis are still poorly understood.

Increasing evidence has confirmed that miRNAs act as novel cellular senescence regulators [26,27]. However, there is little information regarding the potential involvement of miRNAs in regulating the cellular senescence of the corneal endothelium. Therefore, in this study, we identified critical miRNAs that play important roles in regulating the aging of corneal endothelial cells of mice aged 10–13 weeks and mice aged 2 years. These miRNAs include miR-29c, miR-34c, miR-124, miR-695, and miR-32.

Mmu-miR-29c exhibited the greatest increase in expression and has previously been shown to be downregulated in chronic lymphocytic leukemia patients with TP53 abnormalities [28]. Mmu-miR-29c is also associated with strain-specific susceptibility to dietary nonalcoholic steatohepatitis in mice [29]. More importantly, miR-29c induces cell cycle arrest by modulating cyclin E expression [30]. The target genes of miR-29c that were previously validated in other cell types have profound roles in the inhibition of cell proliferation and induce apoptosis by targeting leucine-rich repeat containing 1 (TNFAIP3), beta-site APP cleaving enzyme 1 (BACE1), and T cell lymphoma invasion and metastasis 1 (TIAM1) [30-32]. In contrast, mmu-miR-695 exhibited the greatest decrease in expression in the old mice. However, at present, there are no reports regarding the function of miR-695. Therefore, from the results of this study, we hypothesize that miR-695 may contribute to corneal endothelial cell senescence.

It was reported that miR-34b and miR-34c are targets of p53 and cooperate in the control of cell proliferation and adhesion-independent growth [33]. It is well known that p53 is a critical mediator of the senescence response to several stimuli, such as DNA damage or oxidative stress. Cellular senescence is primarily divided into two subsets based on mechanism. One subset is telomere-initiated replicative senescence and the other subset is telomere-independent stress-induced premature senescence, which is brought about by cellular stress. Therefore, in this study, the differential expression of miR-34c suggested that p53 plays a pivotal role in cellular senescence of corneal endothelium.

Zhu et al. [34] reported that downregulated miRNA-32 expression induced by high glucose inhibits cell cycle progression via PTEN upregulation and Akt inactivation in bone marrow–derived mesenchymal stem cells. Shin et al. [35] found that miR-32 and its target SLC45A3 regulate the lipid metabolism of oligodendrocytes and myelin. As far as we knew, this is the first time we found that miR-32 was involved in cell senescence, especially in corneal endothelium. It has been reported that miR-183 was increased in H_2_O_2_-induced cellular senescence [36,37]. Furthermore, a recent paper showed that Notch, a critical regulator of senescence, is regulated by miR-31 [38].

In addition, miR-124 was found to be involved in the regulation of cell differentiation, cell cycle arrest and apoptosis in neuroblastoma, hepatocellular carcinoma, and medulloblastoma [39]. Huang investigated the pivotal role of miR-124 in neuroblastoma and found that silencing miR-124 induces neuroblastoma SK-N-SH cell differentiation, cell cycle arrest, and apoptosis [40]. Lang reported that miR-124 suppresses cell proliferation in hepatocellular carcinoma by targeting PIK3CA [41]. Moreover, miR-124 regulates early neurogenesis in the optic vesicle and forebrain by targeting NeuroD1 [42]. In this study, miR-124 was found to be upregulated in the corneal endothelium of old mice. We also found that the neurotrophin signaling pathway appeared to correlate with corneal endothelium senescence. Previously published studies suggest that several types of neurotrophin promote the growth of corneal endothelial cells, such as vasoactive intestinal peptide [43,44] and nerve growth factor [45]. Therefore, changes in the neurotrophin signaling pathway may play important roles in corneal endothelial cell senescence.

In conclusion, our study identified the miRNAs that are differentially expressed in the corneal endothelium during aging for the first time, as well as fluctuations in the expression of these specific miRNAs that may be related to age-specific changes. Therefore, our results identify several exciting directions for future research. Each specific miRNA may be a strong candidate for a major study to create a more detailed picture of the pathogenesis of corneal aging. Further studies are needed to investigate the regulatory mechanisms of these aging-associated miRNAs. Understanding miRNA expression and interactions in a tissue such as the cornea may help in understanding the basic and pathophysiological processes of age-related ocular pathologies.

## Appendix 1.

A full list of normalized downregulated microRNA expression in the corneal endothelium of old mice compared to young mice. To access the data, click or select the words “Appendix 1.”

## Appendix 2.

A full list of normalized upregulated microRNA expression in the corneal endothelium of old mice compared to young mice. To access the data, click or select the words “Appendix 2.”
